# Antibody-Dependent Transplacental Transfer of Malaria Blood-Stage Antigen Using a Human Ex Vivo Placental Perfusion Model

**DOI:** 10.1371/journal.pone.0007986

**Published:** 2009-11-24

**Authors:** Karen May, Markus Grube, Indu Malhotra, Carole A. Long, Sanjay Singh, Kishor Mandaliya, Werner Siegmund, Christoph Fusch, Henning Schneider, Christopher L. King

**Affiliations:** 1 Department of Pharmacology, Ernst Moritz Arndt University of Greifswald, Greifswald, Germany; 2 Department of Pediatrics, Ernst Moritz Arndt University of Greifswald, Greifswald, Germany; 3 Center for Global Health and Diseases, Case Western Reserve University, Cleveland, Ohio, United States of America; 4 Malaria Vaccine Development Unit and Laboratory of Malaria and Vector Research, National Institutes of Health, Bethesda, Maryland, United States of America; 5 Pathology Services, Kenyan Ministry of Health, Mombasa General Hospital, Mombasa, Kenya; 6 Department of Obstetrics and Gynecology, University of Berne, Berne, Switzerland; 7 Louis B. Stokes Veterans Affairs Medical Center, Cleveland, Ohio, United States of America; Sabin Vaccine Institute, United States of America

## Abstract

Prenatal exposure to allergens or antigens released by infections during pregnancy can stimulate an immune response or induce immunoregulatory networks in the fetus affecting susceptibility to infection and disease later in life. How antigen crosses from the maternal to fetal environment is poorly understood. One hypothesis is that transplacental antigen transfer occurs as immune complexes, via receptor-mediated transport across the syncytiotrophoblastic membrane and endothelium of vessels in fetal villi. This hypothesis has never been directly tested. Here we studied *Plasmodium falciparum* merozoite surface protein 1 (MSP1) that is released upon erythrocyte invasion. We found MSP1 in cord blood from a third of newborns of malaria-infected women and in >90% following treatment with **acid dissociation** demonstrating MSP1 immune complexes. Using an *ex vivo* human placental model that dually perfuses a placental cotyledon with independent maternal and fetal circuits, immune-complexed MSP1 transferred from maternal to fetal circulation. MSP1 alone or with non-immune plasma did not transfer; pre-incubation with human plasma containing anti-MSP1 was required. MSP1 bound to IgG was detected in the fetal perfusate. Laser scanning confocal microscopy demonstrated MSP1 in the fetal villous stroma, predominantly in fetal endothelial cells. MSP1 co-localized with IgG in endothelial cells, but not with placental macrophages. Thus we show, for the first time, antibody-dependent transplacental transfer of an antigen in the form of immune complexes. These studies imply frequent exposure of the fetus to certain antigens with implications for management of maternal infections during pregnancy and novel approaches to deliver vaccines or drugs to the fetus.

## Introduction

Fetal exposure to allergens or molecules released by various infections during pregnancy has been an area of intense study over the past several decades. The immunological influences of early exposure to these antigens may have profound effects on subsequent susceptibility to allergy, atopy and risk of infection and disease later in life [1]. How and when these antigens cross from maternal to fetal environments may impact on the type of early immune responses and why such responses appear to be restricted to certain antigens. In the current study we use malaria as a paradigm to study the mechanisms of transplacental transfer of antigens because the burden of infection and disease occurs during pregnancy and early childhood.

The greatest susceptibility to malaria is during early childhood, and most deaths due to malaria occur within the first 3 to 5 years of life [2]. Considerable effort has focused on the genetic, immunological, and environmental factors that influence this susceptibility to malaria following birth. Prenatal factors affecting the fetus *in utero*, however, have received comparatively little study [3]. The fetus may be exposed to malaria because pregnant women are at increased risk for infection as compared to nonpregnant women [4]. Malaria infected erythrocytes preferentially sequester in the placenta, **producing an inflammatory responses in the intervillous space, that likely contribute to premature delivery or intrauterine growth retardation by mechanisms that remain poorly understood** [5], [6]. The observation that cord blood mononuclear cells from as many as two thirds of newborns in malaria endemic areas produce cytokines to malaria blood stage antigens [7], [8] suggests malaria-infected erythrocytes [9], [10], [11], [12] and/or their soluble products cross the placenta, where fetal lymphocytes are primed *in utero* [13], [14].

The mechanisms by which the fetus is exposed to malaria blood stage antigens remain poorly understood. Congenital malaria is uncommon and typically occurs in 10% or less of newborns of malaria-infected women [9], [11], [15], [16]. More likely soluble malaria blood-stage antigens released upon rupture of infected placental erythrocytes expose the fetus. Surprisingly, this possibility has received little attention. Only a few studies have reported malaria antigens in cord blood plasma from a small percentage of newborns [11], [13]. The limited ability to detect malaria blood stage antigens with conventional immunoassays may be due to the fact that the antigens are bound into immune complexes [17], [18], [19]. As with many acute and chronic infections, immune complexes (ICs) have been noted in asymptomatic and symptomatic individuals alike [19], [20]. Immune complexes have been detected in the placenta of malaria-infected women [17], but never in cord blood. Surprisingly, little is known about the biochemical nature of these ICs in malaria or what antigens are contained in the complexes [21].

Previous studies have suggested transplacental transport of ICs, most notably after immunization with tetanus toxoid [22], [23], after exposure to common allergens [24], [25], and with administration of non-human insulin in diabetic women [26], [27]. These studies, however, have never directly demonstrated transplacental transfer of antigen bound to antibody. Here we hypothesize that malaria blood stage antigens are transported transplacentally to the fetus as ICs. To test this hypothesis we focused on *Plasmodium falciparum* MSP1, which is a 195-kDa GPI- anchored protein on the merozoite surface, representing the most abundant merozoite surface protein [28]. **We have previously shown frequent fetal (cord blood) reactivity to MSP1 in a malaria endemic area of Kenya** **[14]**. MSP1 undergoes a series of proteolytic cleavages during merozoite invasion of erythrocytes. The final cleavage of the C-terminal 42kD portion of MSP1 (MSP1_42_) releases a soluble fragment [29], with the most C-terminal 19kD portion being retained on the surface, and later carried into the erythrocyte during invasion [30]. Here we first evaluate whether C-terminal fragments of MSP1 occur in cord blood of offspring of malaria-infected women and whether they are complexed with antibody. Next we study whether recombinant MSP1_42_, either alone or in the presence of anti-MSP1 antibody, is transplacentally transported from the maternal to fetal circulation, using a dual *ex vivo* perfusion model of the human placenta.

## Materials and Methods

### Human Plasma Samples

Eighteen paired samples of both maternal peripheral blood and intervillous placental blood were obtained from *P. falciparum* -infected women residing in a malaria holoendemic area in Kwale District, Coast province, Kenya at delivery as described [11]. We also examined cord blood from 20 women who did not have evidence of malaria at delivery as well 15 cord blood samples from newborns that delivered from healthy women in Cleveland, OH. The maternal intervillous samples were obtained by cannulation of the intervillous space with a 16-gauge needle through the basal plate of the placenta. Cord blood from their offspring was collected at delivery by cannulation of the umbilical vein proximal to the point where the umbilical cord had been clamped. The presence of *P. falciparum* infection was assessed by blood smear and/or real-time quantitative PCR of maternal and cord blood samples as described [11]. All samples were collected following written informed consent of participating women. The Scientific and Ethical Review Committees of the Kenyan Medical Research Institute and the Institutional Review Board of Case Western Reserve University approved the study. Pooled human plasma containing anti-MSP1 immunoglobulin (endpoint titer to 1∶256,000, by ELISA) was obtained from expired anonymous units of blood collected at the Coast Province Blood Bank in Kenya, all of which were negative for HIV and Hepatitis B antibodies. The blood units were blood smear and PCR negative for malaria. **There was no MSP1 detected in the plasma, and not complexed to immunoglobulin**.

### Measurement of MSP1 and MSP1 Immune Complexes in Human Plasma and Placental Perfusate

A summary of the different assays used to detect MSP1 and antibodies directed against MSP1 are summarized in Table 1. Details of the assays are provided in the legend. All assays were performed with a volume of 100ul using Immulon 4 ELISA plates (Dynatech, Inc). Plates were then washed (PBS-0.05% Tween) and blocked with PBS+3%BSA for 1 hr at 37°C, prior to adding the test samples, which were incubated overnight at 4°C. **The authors generated the polyclonal rat and rabbit anti-sera directed to MSP1_42_**. The sera, either rabbit or goat **as secondary antibodies**, were obtained from Jackson ImmunoResearch (West Grove, PA) and diluted in PBS to 1∶1000 then added to plates for 2h at 37°C. Following washes, 5-bromo-4-chloro-3-indolyl phosphate/nitro blue tetrazolium alkaline phosphatase substrate solution (Sigma) was added and samples read by a VERSA_max_ microplate reader (Molecular Devices, Sunnydale, CA) at OD_422_ µm. Assay 1 measured MSP1 in plasma samples and perfusate samples. **Assay 2 measured anti-MSP1_42_, not bound to MSP1_42_, in serum and perfusate samples from the maternal and fetal circulation. Assay 3 measured antigen-antibody complexes using biotinlyated rMSP1_42_ (FVO allele [bMSP1_42_]). Assay 4 measured antibodies that bound bMSP1_42_ in maternal and fetal circulation, and anti-MSP1_42_**.

**Table 1:** Assays used to detect MSP1 and antibodies to MSP1.

Assa No.	Assay for	Material Assayed	Capture Ab (Ag)	Detection	Conjugate
1^a^	MSP1	Plasma/perfusate	Affinity Purified Rabbit anti-MSP1_42_ (1∶1000 dilution)	Rat anti-MSP1_42_ (1∶1000 dilution)	Goat α-rat alkaline phosphatase (AP)
2^b^	Anti-MSP1_42_	Plasma/perfusate	rMSP1_42_ (0.5 mcg/ml)	Goat anti-human IgG (H+L)-AP (1∶1000 dilution)	NA
3^c^	Biotinylated rMSP1_42_ (bMSP1_42_)	Perfusate	Streptavidin (5mcg/ml)	Rabbit αMSP1_42_	Goat anti-rabbit AP
4^d^	Anti-MSP1 bound to bMSP1_42_	Perfusate	Streptavidin (5 mcg/ml)	Goat anti-human IgG (H+L)-AP (1∶1000 dilution)	NA

a Polyclonal anti-MSP1_42_ was resuspended in carbonated-bicarbonated buffer (pH 9.6) and **used to coat the ELISA plates as described in the materials and methods section. As a standard, recombinant MSP1_42_ was resuspended in the same media used for the test samples (i.e. plasma from N. American subjects or perfusates) and added to the wells coated with anti-MSP1_42_ at serial two-fold dilutions starting at 4 ng/ml**. Sensitivity of the assay was approximately 200 pg/ml.

b Plates were coated with rMSP1_42_ in PBS.

c Plates were coated with streptavidin in PBS (Pierce Biotechnology Inc). Standards used for this experiment were bMSP1_42_ or pooled human plasma containing anti-MSP1 (25%) combined with bMSP1_42_ as described in the text. The assay had a sensitivity ∼175–200 pg/ml **to detect the bMSP1_42_ in the samples**.

d Perfusate was added at 1∶5 dilution to wells coated with streptavidin and incubated overnight at 4°C. Plates were washed thoroughly (6 times) with 0.1% Tween as described in the text to remove any unbound antibody prior to adding goat anti-human IgG. Control experiments using MSP1_42_ incubated with normal serum had <0.15 OD and the mean+2 SD was subtracted from background described in figure 4.

To dissociate antigen-antibody complexes we used an acid dissociation protocol that has been used for several blood-borne viruses [31], [32]. Patient plasma samples or perfusate were either diluted 1∶3 in phosphate-buffered saline (PBS) (nondissociated samples) or 1∶2 in dissociation buffer (1.5 M glycine-HCl [pH 2.0]). The antigen-antibody complexes were dissociated for 30 minutes at 37°C and the reaction was stopped by addition of 1 volume of neutralization buffer (1.5 M Tris-HCL [pH 9.7]) to achieve an end dilution of 1∶3 for the sample. An additional 2 volumes of PBS were added for a final dilution of 1∶5. The sample was then immediately measured for MSP1 using assay 1.

### Measurement of Placental Alkaline Phosphatase (PLAP)

PLAP level was measured by use of a method of Kaneda et al [33] and modified by Kwiek, et al [34]. The PLAP level in cord and maternal blood was determined by interpolation from a standard curve of purified human PLAP (Sigma) fit to quadratic equation.

### Placental Donors

Six human term placentas (gestational age 38–40 weeks, placenta weight 648–918 g) were obtained from uncomplicated singleton pregnancies delivered by caesarean section or vaginal delivery. The local ethical review committee at the University of Greifswald approved the study. **Prior to delivery, women gave written informed consent for use of their placentas for the study**.

### Placenta Perfusion Model and Experimental Design

**Figure 1:**
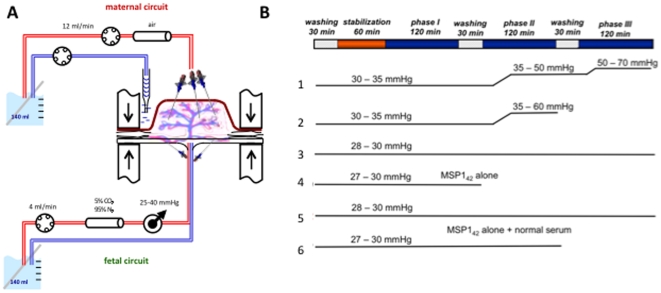
Perfusion model and experimental design. Panel (A) shows a diagram of the *ex vivo* placenta perfusion model as performed in the study. Details of the assay are described in the methods section. **Of note 3 probes on the maternal side cannulate the intervillous space and on the fetal side one probe each cannulate the artery and vein**. Panel (B) shows the experimental design for each of the 6 placentas studied. A complete experiment comprised 3 phases. Phase I involved addition of rMSP1_42_ to the maternal circulation with samples removed from the maternal and fetal circulation every 30 minutes until the completion of the phase. Phase II and phase III involved addition of rMSP1_42_ previously mixed with 10% or 25% human plasma containing anti-MSP1 antibodies to form ICs. The perfusion pressures recorded throughout the experiment are shown. A significant rise in perfusion pressure indicates poor placental flow of medium through the vasculature. If pressures rose too quickly as in placenta 2, the experiment was terminated. Placenta 4 was terminated early to examine whether MSP1_42_ without specific antibody could be detected by immunohistochemistry in the villous stroma. Placenta 6 included MSP1_42_ with 25% human plasma from individuals that have never resided in a malaria endemic area and did not have a phase with lower serum concentrations.

The dual circulation human placenta perfusion model [35] included cannulation of the chorionic artery and the corresponding vein of a suitable cotyledon, to perfuse the fetal side within 20 minutes of delivery (Figure 1A). The maternal side was perfused by three blunted cannulas, which were introduced into the intervillous space. The perfusion medium consisted of NCTC 135 (SIGMA-Aldrich, Steinheim, Germany) in Earl's buffer (1∶3, v/v), 4% albumin (PAA Laboratories, Linz, Austria), 0.2% glucose (Merck, Darmstadt, Germany), 1% dextrane 40 (Carl Roth, Karlsruhe, Germany), 2,500 units/l heparin (Braun, Melsungen, Germany), and 250 mg/l clamoxyl (Ratiopharm, Ulm, Germany). To mimic the intrauterine conditions, two gas exchange devices (Mera Silox-S 0.3, Senko Medical Instruments, Tokyo, Japan) were connected; the fetal perfusate was equilibrated with 95% nitrogen and 5% carbon dioxide, whereas an atmospheric gas mixture was used for the maternal side. The flow rate was 12 ml/min in the maternal and 4 ml/min in the fetal circuit, resembling physiologic flow rates. The fetal arterial pressure was maintained between 25 and 40 mmHg and the total perfusion volume in each circuit was 140 ml.

At the beginning of all experiments, the placenta was perfused for 30 minutes without return of the perfusate to the circulation (open loop), to remove blood and metabolites from the two placental circulations. This was followed by a closed-loop perfusion (recycling of perfusate) for 60 min to equilibrate the placenta and to identify leaks or shunts between maternal and fetal perfused circulation. Experiments were terminated for fetal perfusion pressure above 50 mmHg, for loss of perfusate>4 ml/h from the fetal circulation, and for cases of mismatch of maternal-fetal circulation as measured by the antipyrine permeability (i.e. lower levels of permeability, <0.04 ml×min^−1^×g^−1^, Table 2).

**Table 2:** Permeability of antipyrine, creatinine, erythropoietin and viability characteristics of perfused placentas used in the studya.

Protocol	1^b^	2	3	4	5	6
glucose consumption (µmol×min^−1^×g^−1^)	0.336^c^±0.106	0.186±0.109	0.189±0.062	0.369	0.267±0.010	0.385±0.069
lactate production (µmol×min^−1^×g^−1^)	0.461±0.100	0.387±0.097	0.490±0.062	1.151	0.418±0.106	0.808±0.289
antipyrine permeability (ml×min^−1^×g^−1^)	0.054±0.028	0.08±0.002	0.166±0.064	0.129	0.120±0.029	0.169±0.024
creatinine permeability (ml×min^−1^×g^−1^)	0.021±0.005	0.013±0.003	0.041±0.004	0.047	0.038±0.009	0.049±0.001
erythropoietin permeability (ml×min^−1^×g^−1^)	0.00	0.00	0.00	0.00	0.00	0.00
hCG release (mU×min^−1^×g^−1^)	69. 17	78.57	31.10	307.66	102.03	139.84

a All values shown fall within the range of values for previous placental perfusion studies without any experimental interventions [38].

b Experiment number (each represents a complete perfusion experiment with different placentas.

c Means±SD are shown for all phases of the experiment. For placenta 4 only one phase is shown.

After the initial equilibration of the placenta for 60 min in which the medium was exchanged in both circuits, the actual experiment consisted of three phases lasting 120 min each (Figure 1B). All media were mixed one day before the experiment and incubated overnight at room temperature. Between experimental phases, a 30 min “wash-out” using open perfusion with fresh media was performed on both the maternal and fetal circulations, to wash out any antigen and/or antibody. For phase I, rMSP1_42_ was added to the maternal side only at a final concentration of 1 µg/ml, and both the maternal and fetal perfusates were sampled every 30 minutes as described above. For phase II fresh maternal perfusate was modified to include 1 µg/ml MSP1_42_ and 25% immune plasma containing anti-MSP1 antibodies, to form ICs described above. For phase II the fetal perfusate medium contained 25% fetal calf serum (FCS, without MSP1_42_) to maintain a similar oncotic pressure for fetal and maternal circulations. For phase III, the maternal circulation contained 1 µg/ml MSP1_42_ and 10% immune plasma containing anti-MSP1 antibodies, and the fetal circulation contained 10% FCS without MSP1_42_. (Of note, phase II and phase III were reversed for experiment 5). Two control experiments were performed, consisting of i) perfusion with MSP1_42_ in the absence of antibodies, with early termination to examine histological sections (No. 4) and ii) perfusion with MSP1_42_ preincubated with 25% plasma from individuals who had never traveled to malaria endemic areas and therefore lacked anti-MSP1 antibodies (No. 6). Initially, experiments with placentas Nos. 1 and 2 were terminated early because of an increase in fetal perfusion pressure following addition of immune plasma (phase 2). Experiment No. 2 had to be terminated early. In subsequent experiments plasma was first dialyzed against phosphate buffer saline (PBS) with a MW cut-off of 25 kD, which eliminated the increase in perfusion pressures during the course of the experiment. Data on viability as assessed by consumption of glucose and production of lactate and human chorionic gonadotropin (hCG) during the course of the experiments as well as permeability as measured by antipyrine (0.4 µM), creatinine (1.3 µM) and erythropoietin (1.0 ug/ml), which were also added in all three experimental phases in the maternal circuit, were consistent with previous studies (Table 2).

### Sample Collection

One ml aliquots were obtained from both the fetal and maternal circulations at the beginning, and every 30 minutes thereafter. Prior to removal of medium the volumes were checked in each reservoir for evidence of perfusate leak from the maternal to the fetal circulation. Placental tissue samples were randomly taken from an unperfused area before the beginning of each experiment and from the perfused cotyledon at the completion of the whole experiment. Samples were immediately cryopreserved or fixed in 4% paraformaldehyde and embedded in paraffin.

### Assays for Glucose, Lactate, Creatinine, Antipyrine, hCG and Erythropoietin Biometrical Evaluation

Glucose, lactate, creatinine, antipyrine and hCG concentrations were measured as described recently [36]. Erythropoietin was measured using the Human Erythropoietin Quantikine IVD ELISA Kit (R&D Systems, Minneapolis, USA) according to the supplier's instructions. Permeability of antipyrine and creatinine was calculated according to the equation P = C_fet_/(weight_cot_×[AUC_mat_−AUC_fet_]) with C_fet_ to be the concentration in the fetal circuit at the end of perfusion, AUC_mat_ the area under the concentration-time curve (AUC) in the maternal circuit, AUC_fet_ the AUC in the fetal circuit, and weight_cot_ the wet weight of the cotyledon [37]. The hCG release was calculated as described [38]. Means ± standard deviations (SD) are given. Sample statistics were done using the Wilcoxon test.

### Immunohistochemistry

#### Paraffin fixed sections of the placenta were sectioned at 2 µm thickness, then deparaffinized and rehydrated

Sections were boiled for 10 min with citrate buffer (pH 6.0, 1.8 mmol/L citric acid, 12.2 mmol/L sodium cirate) before blocking with 5% fetal calf serum for 2 hours. MSP1_42_ was detected using 1∶50 dilution of rat anti-MSP1_42_ polyclonal Ab **(the same antibody used for antigen capture assay in** **Table 1** **)** followed by a AlexaFluor 488 conjugated goat anti-rat secondary antibody (1∶200, Invitrogen, Carlsbad, Ca, USA). A mouse anti-CD68 (mAb Kp1, 1∶25, Santa Cruz Biotechnology Inc., Santa Cruz, CA) and a rabbit anti-CD31/PECAM-1 (1∶200, Santa Cruz Biotechnology Inc.), followed by an AlexaFluor 568 conjugated goat anti-rabbit and anti-mouse secondary antibody (1∶100, Invitrogen), respectively, were used for staining of macrophages/Hofbauer cells (anti-CD68) and endothelial cells (anti-CD31). Nuclear counterstaining was performed using TOTO-3-iodide (Invitrogen) added to the mounting medium (dilution 1∶2000, DakoCytomation, Glostrup, Denmark). Co-localization of markers was examined using a laser scanning confocal microscope. **Immunohistochemistry was performed on samples from all 6 placentas used**.

### Statistical Analysis

**Figure 2:**
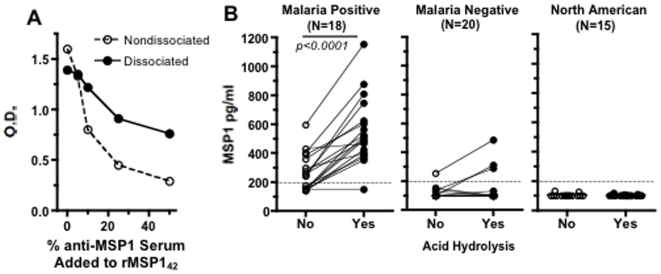
Detection of MSP1_42_ complexed to antibodies in cord blood of plasma from Kenyan newborns. Adding varying concentrations of plasma containing anti-MSP1_42_ with recombinant MSP1_42_ impaired detection of MSP1_42_ using an antigen capture ELISA (assay 1) indicating formation of ICs. Dissociation of ICs partially restored the ability to detect MSP1_42_ **(A)**. Panel **(B)** shows the presence of MSP1 in cord blood plasma of offspring of malaria infected Kenyan women detected by ELISA (assay 1) before and after dissociation of ICs (connecting lines). Each point represents the average of two independent assays run on the same sample. Variation for the repeat assay for each sample was <16%. **The p value shown in** **figure 2B** **comparing samples before and after acid dissociation was performed was using a paired** ***t*** **-test**.

Comparisons of MSP1_42_ levels were performed by Student's *t*-test of log-transformed values. When appropriate, paired *t*-tests were performed (i.e. Figure 2B).

## Results

### Circulating MSP1 and Immune Complexes in Cord Blood

Soluble MSP1 was present in the blood of pregnant women infected with malaria and in cord blood of their newborns (Table 3). The presence of MSP1 in cord blood is unlikely to have occurred by admixture with maternal blood at delivery. We measured the levels of placental alkaline phosphatase (PLAP) in both maternal intervillous and cord blood in an initial group of 5 paired samples. PLAP is produced in high levels by the syncytiotrophoblast (STB) and released solely into the maternal compartment of the placenta [33], and thus is used as a marker of maternal-fetal transfusion during the peripartum period [11], [33], [34]. PLAP levels were undetectable in 4 of 5 cord blood samples and low in the remaining one (Table 3). **We cannot exclude the possibility that MSP1_42_ detected in cord blood for case 3 may have occurred because of admixture from maternal blood**. By contrast PLAP levels were markedly higher in maternal intervillous blood, indicating little or no peripartum maternal to fetal transfusion. There was no correlation between levels of MSP1 in maternal and cord blood **(r^2^ = 0.13, p = 0.19)**. Thus the MSP1 detected in cord blood was unlikely to have resulted from admixture with maternal blood.

**Table 3:** MSP1 is present in cord blood of women with malaria at delivery.

*Case*	*Cord Blood*		*Maternal Peripheral and/or Intervillous blood* a	
	*MSP1 (pg/ml)*	*PLAP* b *(ng/ml)*	*MSP1 (pg/ml)*	*PLAP (ng/ml)*
1	272	<20	936	361
2	431	<20	592	1,107
3	297	37	669	232
4	389	<20	1,256	1,845
5	241	<20	520	688

a All mothers were malaria infected at the delivery as shown by either PCR and/or blood smear. **Cases 1–4 represent intervillous blood samples and case 5 maternal peripheral blood**.

b Placental alkaline phosphatase.

We next examined whether antibody bound to MSP1 impairs its detection by ELISA (i.e. assay 1, see table 1). Addition of 1 ug/ml of purified rMSP1_42_ in PBS with varying concentrations of pooled plasma containing anti-MSP1 antibodies from malaria exposed (but not actively infected) individuals reduced detection of antigen in a dose-dependent fashion (Figure 2A). This suggests formation of antigen-antibody complexes. Dissociation of these complexes with dissociation buffer partially restored the ability to detect MSP1. Of note, dissociation buffer treatment of rMSP1_42_ without antibody resulted in partial reduction in antigen detection, probably due to denaturation of some epitopes.

In addition to the 5 initial paired maternal and cord blood samples, we examined an additional 13 paired maternal samples infected with malaria **(5 intervillous and 8 peripheral venous blood)** at delivery, to assess whether MSP1 may be complexed to antibody, thereby impairing its detection. Without treatment of samples with dissociation buffer, MSP1 was detected in 5 of 13 cord blood plasma samples (Figure 2B); following treatment with dissociation buffer, MSP1 detection increased to 12 of 13 cord samples (Figure 2B). **Of note, the 5 cord blood samples shown in** **Table 3** **, acid dissociation also enhanced detection of MSP1_42_ in 4 samples (also shown in** **Figure 2B** **)**. MSP1 was detected in maternal samples in 7 of 13 individuals before, and in all samples following treatment with dissociation buffer (data not shown). There was no correlation between MSP1 levels in paired maternal and cord blood. Free antibody to MSP1_42_ of varying titers was present in all 18 paired maternal cord blood samples (data not shown). **There was no relationship between maternal anti-MSP1_42_ antibody levels and the presence and levels of MSP1_42_ in cord blood, either before or after acid dissociation (data no shown)**. In cord blood of malaria negative women at delivery, 1 of 20 (5%) and 3 of 20 (15%) of cord blood samples had detectable MSP1 before and after acid dissociation respectively, indicating the persistence of antigen even after the parasitemia is cleared (Figure 2B). No MSP1 was detected in cord blood from North American newborns. Thus, MSP1 is frequently found in malaria-infected women's peripheral and/or intervillous placental blood and in cord blood from their offspring. MSP1 is often complexed to antibody.

### Transplacental Transport of MSP1_42_ Using the *Ex Vivo* Placenta Perfusion Model

**Figure 3:**
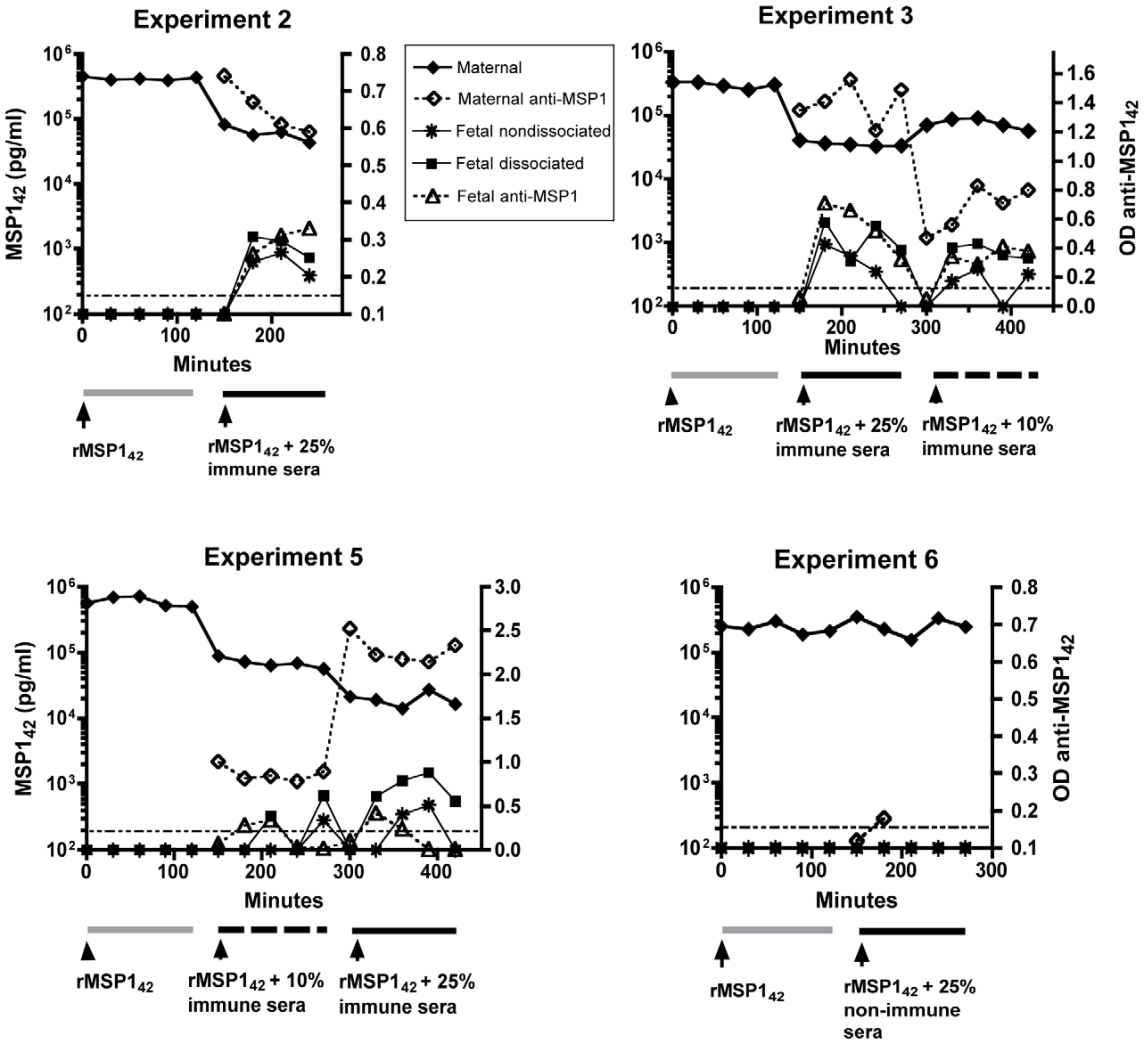
Measurement of MSP1_42_ and free anti-MSP1 antibodies in maternal and fetal circulation with the placental perfusion model. The levels of MSP1 in the fetal perfusate were measured before (nondissociated) and after treatment with dissociation buffer (dissociated) during the 3 phases of the experiment. **The differences in levels of MSP1_42_ detected in the fetal perfusate between phases II and III (placentas 3 and 5) correlated with percentage of plasma containing anti-MSP1 antibodies added to the maternal compartment**. Each point represents the mean value performed in triplicate. The variation between replicate samples was <10%. **Assay 1 was used to measure free MSP1 and assay 2 for free anti-MSP1 antibodies (see** **table 1** **)**.

Since none of the cord blood samples identified as containing MSP1 had evidence of malaria parasites (blood smear and PCR negative) at delivery, we postulated that soluble MSP1 crosses transplacentally from maternal to fetal circulation during gestation. To test this hypothesis we used a dual *ex vivo* placental perfusion model (Figure 1A) to examine whether transplacental transfer of soluble MSP1 can occur alone or in complex with specific antibodies. Addition of rMSP1_42_ alone to the maternal circulation failed to show transplacental antigen transfer (i.e. no MSP1 was detected in the fetal perfusate within the first 2 hours in any experiment, Figure 3). Transplacental transfer of MSP1_42_ occurred only when the antigen was previously incubated with plasma containing specific anti-MSP1 to form antigen-antibody complexes (described in Figure 2A), as shown for placentas Nos. 2, 3 and 5 (Figure 3). Transplacental transport increased with higher concentrations of plasma (Nos. 3 and 5). There was no transplacental transport of MSP1 mixed with plasma that did not contain specific antibodies to MSP1 (No. 6). Transplacental transport occurred quickly, in that antigen was detected 30 minutes following addition of antigen plus anti-MSP1 antibody to the maternal circulation, although the time to peak levels of MSP1_42_ detection varied among perfused placentas. Between 0.3 and 0.9 percent of total MSP1_42_ was transferred from maternal to fetal circulation during the course of the experiments using modifications of the permeability equations. Quantification of antigen by ELISA, however, can be affected by the presence of IC, as well as partial denaturation of antigen with dissociation buffer. To further demonstrate specific transplacental transfer of MSP1_42_ and absence of leaks in the placenta, 1 ug/ml of erythropoietin, a protein of similar size (34 kD), was added to the maternal perfusate in each phase of all experiments. Erythropoietin was never detected in the fetal perfusate (Table 2). This is consistent with results previously shown in this system [39]. Thus transplacental transport of rMSP1_42_ is selective and requires specific antibody.

**Figure 4:**
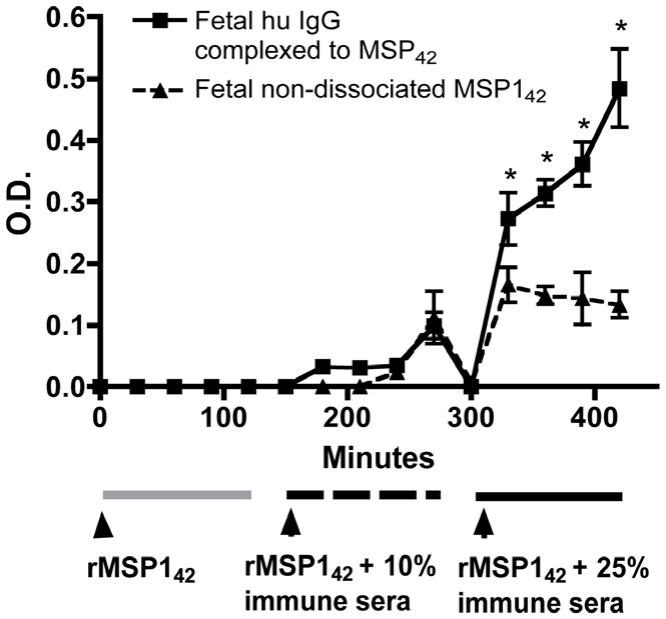
The presence of human IgG complexed to rMSP1_42_ in fetal perfusate. rMSP1_42_ was biotinylated (bMSP1_42_) and thus the configuration of ELISA differed from that described in Fig. 3, e.g. streptavidin was used to capture bMSP1_42_ followed by anti-human IgG (assay 3, see table 1, squares). Nondissociated MSP1_42_ levels were also measured (assay 2, see table 2, triangles). The assay was performed for placenta 5. Values indicate means±SD of replicate assays (N = 3). Asterisks indicate significant differences from P<0.05 to P<0.001 between samples. **Assay 3 was used to measure non-dissociated bMSP1_42_ and assay 4 for human IgG complexed to rMSP1_42_ (see** **table 1** **)**.

As evidence of transplacental transfer of MSP1_42_ occurring in the form of ICs, we noted that more MSP1 in fetal perfusate was detected following treatment with dissociation buffer, indicating that at least some MSP1 was bound to antibody (Figure 3). We also directly observed human IgG antibody bound to rMSP1_42_ in the fetal perfusate. This was accomplished by using biotinylated rMSP1_42_ in the experiment with placenta No. 5 (Figure 4). Biotinylation of rMSP1_42_ permitted a modified MSP1_42_ ELISA (i.e. assay 4, table 1) whereby high affinity binding to streptavidin followed by a more stringent washing protocol allowed removal of non-specific binding of human IgG. Of note, a similar assay was performed on non-dissociated samples with subclass-specific IgG. The predominant antibody bound to bMSP1_42_ in the fetal perfusate was IgG1 and some IgG3, but no IgG2 or IgG4 (data not shown).

Free anti-MSP1 antibody was also detected in the fetal circulation after addition of immune plasma in the presence of antigen to the maternal circulation (Figure 3), but levels were lower compared to that in the maternal circulation. This suggests that either free specific antibody crosses the placenta or dissociates from antigen once in the fetal circulation.

### Immunolocalization of MSP1 in the Placenta

**Figure 5:**
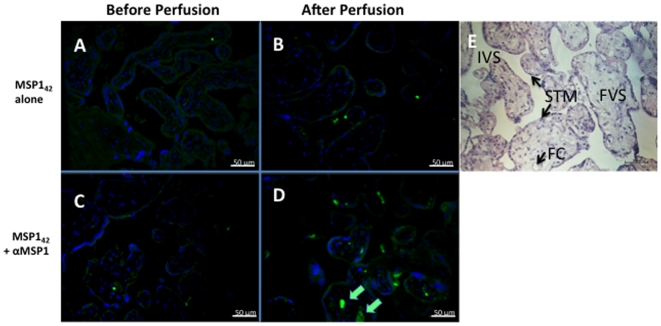
Immunohistological localization of MSP1_42_ in the villous stroma before and after perfusion. Panel (A,C) show before and panels (B,D) after perfusion with MSP1_42_ in the absence of anti-MSP1 (No. 4; A,B) and following addition of anti-MSP1 (No. 5; C,D). The syncytiotrophoblastic membrane (STM) is shown in light green with strings of nuclei (blue). MSP1_42_ has intense green staining only in the villous stroma of No. 5 following perfusion (indicated by arrows, D). **Panel E shows a hematoxylin and eosin stained section from the same placenta for reference. The labels are: IVS – intervillous space, FC – fetal capillary, STM - syncytiotrophoblastic membrane, FVS – fetal villous stroma. All sections are at 200× magnification**. The staining with pre-bleed sera from rats used for making the anti-rMSP1_42_ polyclonal sera was identical to that shown in panels A and C (not shown).

**Figure 6:**
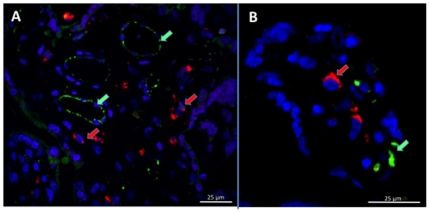
MSP1_42_ does not co-localize with placental macrophages (Hofbauer cells). Panel **(A)** shows MSP1_42_ localizes to fetal vascular structures (green staining, indicated by green arrows) and not with placental Hofbauer cells (CD68 positive cells, red staining indicated by red arrow). Panel **(B)** shows a greater magnification of the villous stroma. **The results are shown for placenta 3**. No co-localization of CD68 positive cells with MSP1_42_ was observed for all 4 placentas examined. **The left had panel at 400× and right at 800×**.

**Figure 7:**
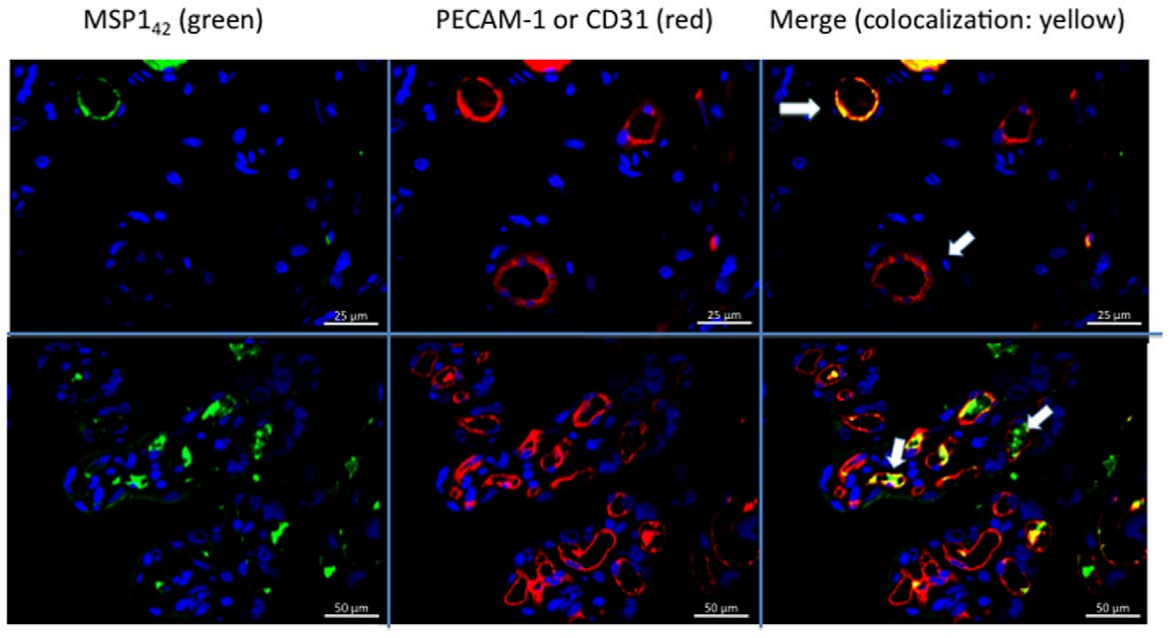
MSP1_42_ co-localizes with fetal endothelial cells following perfusion. MSP1_42_ (green staining) co-localizes with CD31 (PECAM-1, red staining) indicating its presence in and around the fetal vascular endothelial cells (upper and lower panels for placentas 1 and 5 respectively). Similar co-localization was observed for placentas 2 and 3 (not shown). **The upper panels are at 400× and lower at 200× magnification**.

**Figure 8:**
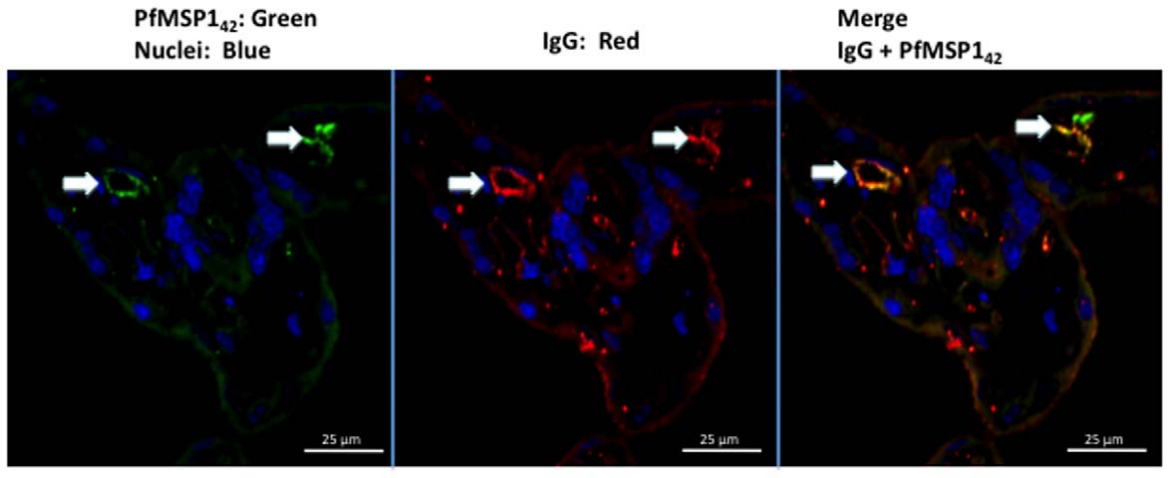
MSP1_42_ co-localizes with human IgG in structures consistent with fetal endothelial cells. This suggests MSP1_42_ may be complexed with IgG. **The results are shown for placenta 3**. **Sections are at 400× magnification**.

We obtained placental biopsies prior to and following perfusion for every placenta. Immunohistochemical localization of MSP1_42_ in the placental villous stroma was not detectable in the absence of specific antibody to MSP1 (Figure 5B), or with non-specific antibody (not shown). By contrast in all experiments in which transplacental transfer of MSP1_42_ occurred in the presence of anti-MSP1, MSP1_42_ was detected in the placental villous stroma (Figure 5 D). **Placental biopsies obtained prior to perfusion (** **Figures 5A and 5C** **, controls) showed slight non-specific staining of STM with the rat anti-MSP1 Ab, but no detection within the placental villous stoma**. Within the stroma MSP1_42_ did not co-localize with placental macrophages (i.e. CD68 positive Hofbauer cells, Figure 6), but accumulated adjacent to or within endothelial cells of fetal vessels (i.e. co-localization with CD31 Figure 7, upper and lower panels). Importantly, MSP1_42_ co-localized with human IgG in and around the endothelial cells of fetal vessels (Figure 8).

## Discussion

There is considerable evidence that dietary antigens [24], [40], allergens [25], [41], [42], [43] and parasite antigens [11], [44] transfer from the maternal to fetal environment *in utero*. This presumably occurs transplacentally or by the fetus swallowing or inhaling antigens in the amniotic fluid. However, the frequency, mechanisms, and selectivity of antigen transfer are poorly understood. Cord blood from offspring of malaria-infected women often acquires T and B cell responsiveness to malaria blood stage antigens, thus raising the question as to how the fetus becomes regularly exposed to these antigens. Generally the placenta provides an effective barrier in preventing exposure of the developing fetus to the harmful effects of pathogens or circulating proteins in the maternal circulation, except those required for fetal growth and development through active transport mechanisms. Occasionally maternal-fetal microtransfusions occur during pregnancy resulting in congenital malaria [11], however the frequency of such infection is too low to account for the proportion of newborns who show immune priming to malarial antigens. This suggests that soluble products of malaria blood stage infection must cross the placenta with some frequency. Here we show that the most abundant malaria blood stage antigen, MSP1 [28], is present in cord blood of offspring of malaria-infected women. The dissociation of ICs in cord blood samples markedly increases the proportion with detectable MSP1. Thus MSP1 may be transported across the placenta bound to antibody.

The placenta has a receptor-mediated capacity to actively transport immunoglobulin G (IgG) from the maternal to fetal circulation [23], [45], [46]. Transplacental transport of IgG begins in the second trimester and increases throughout gestation, with most transport occurring late in pregnancy [47]. Prior studies suggest that IgG may act as a vehicle to carry proteins across the placental barrier from the maternal to the fetal side. Normally human insulin does not cross the placenta [48]. Earlier studies from pregnant diabetic women treated with animal insulin, however, noted that some offspring with macrosomia also had animal insulin in their cord blood [26], [27]. Antibody production can be stimulated by animal insulin, and of note cord blood levels of animal insulin were directly related to maternal levels of insulin antibodies. This suggests a role for insulin bound to antibody in transplacental transport. Another study examining transplacental transport of tetanus toxoid (TT) antibodies using the *ex vivo* placental perfusion model reported tetanus antigen in both the maternal and fetal perfusate. The ratio of TT antigen to TT-specific antibody in the maternal perfusate closely correlated to that observed in the fetal circulation, suggesting coupling of antigen transfer to the specific transport of antibody [22], [23]. Immune complexes with cat allergens have also been noted in cord blood [41], [42].

Using the same *ex vivo* placenta perfusion model, we show that transplacental transfer of rMSP1_42_ required the presence of specific antibodies bound to MSP1. The increased ability to detect MSP1 following dissociation of the ICs, direct detection of human IgG complexed to MSP1_42_ in the fetal perfusate, and co-localization of MSP1_42_ with human IgG in the endothelium of the fetal capillaries suggest that antigen complexed with antibody crossed the placenta to the fetal circulation. Free antibody to MSP1_42_ was also found in the fetal circulation; whether this represents transplacental transport of antibody alone, dissociation of IC after transport to the fetal circulation, or both, is unclear. The ability to detect MSP1_42_ in the maternal plasma **samples or maternal perfusate** without acid dissociation may represent excess free antigen relative to specific antibody. The presence of free MSP1_42_ in the fetal and maternal perfusate may also represent recognition of epitopes on MSP1_42_ by the rat and rabbit antisera that are not bound by specific human antibodies. **Of note there was a decrease in the levels of MSP1_42_ and anti-MSP1 in the fetal perfusate with time suggesting possible protein catabolism or binding within the fetal circulation, although the mechanisms remain unclear**.

The exact mechanism by which IgG facilitates the transplacental passage of a particular antigen is unclear. It has been postulated that the syncytiotrophoblast (STB) takes up ICs by micropinocytosis, which then accumulate in the endosomal compartment. The lower pH in endosomes facilitates the binding of IC to MHC-class I related I Fc receptors of the neonate (FcRN). Immune complexes that remain bound to FcRN do not undergo degradation. The endosomes are then recycled to the basolateral surface of the STB where the higher pH releases the ICs from FcRN. If antigen dissociates from the antibody while in the endosome it is shunted to the lysosome for degradation [46]. Thus survival of antigen in the endosome depends on its affinity to antibody at acidic endosomal pH, the degree to which the endosome can acidify, and the endosomal transit time.

Following deposition of antigen and antibody in the basolateral surface of STB, they are somehow transported through the villous stroma, perhaps by passive diffusion, until reaching the endothelial cells where again a specific transport system mediates transendothelial cell transfer to the fetal circulation. The fetal endothelial cells are too tightly apposed to each other to allow passive diffusion of such large molecules [49]. The exact mechanisms by which human IgG, much less antigen complexed to antibody, passes through fetal endothelial cells remain unknown. The FcRN may also mediate transfer, however FcRN is only occasionally or weakly expressed in fetal vessel endothelium in the placenta [50], [51], [52]. Another receptor in fetal endothelial cells, FcγRIIb, has been found to colocalize with human IgG suggesting a role in its transport [53]. Placental macrophages or Hofbauer cells in the villous stroma expressing FcγRI, RII, and RIII, are thought to bind ICs, forming part of the protective barrier of the placenta between the mother and the fetus [54], [55]. Using laser scanning confocal microscopy, we co-localized the malaria antigen within and adjacent to endothelial cells, but not in the STB or with Hofbauer cells. The failure to observe malaria antigen in STB may simply reflect the rapid transport of antibody and antigen across this membrane. However, the lack of co-localization of the MSP1_42_ with Hofbauer cells is not compatible with the notion that these cells capture ICs (or at least, the ICs formed by MSP1_42_). A number of questions remain as to exactly how antigens cross to the fetal circulation. Is there intracellular dissociation of the IC from FcRN in STB and/or endothelium, with routing of free antigen by apparently unclear mechanisms into the fetal circulation? Alternatively, whether there is “trapping” of ICs in the villous stroma and passage of excess ICs into the capillaries, with subsequent release into fetal blood, needs to be addressed in future studies.

Notable was that only some fetal endothelial cells demonstrated immunohistochemical localization of MSP1_42_ and/or IgG. It is possible that there may be variation in the circulation within the perfused placenta such that some endothelial cells may have more rapidly transported the antigen and/or IgG, while others did more slowly, resulting in heterogeneity of immune localization.

Pregnant women often become infected and ICs can form during the course of these infections. If ICs cross to the fetal circulation as easily as suggested in the current study, the cumulative effect on the fetus would presumably be detrimental. Thus there is likely some selectivity in the transplacental trafficking of IC, perhaps based on IC structure. Immune complexes vary depending on the valency of bound ligands. A recent study showed that multimeric IC that bind to FcRN in the endosomes of dendritic cells are targeted to lysosomes and undergo degradation, whereas monomeric IC do not undergo such a fate [56]. Whether a similar mechanism occurs in the STB is unclear. It is conceivable that MSP1_42_ forms monomeric or dimeric IC because this molecule has few B cell epitopes [57]. By contrast other malaria antigens with multiple B cell epitopes may form multimeric IC that rarely transfer transplacentally.

Placental malaria can cause a number of pathologic changes in the placenta [6] and these changes can reduce transplacental transfer of specific IgG immunoglobulins [58]. In addition repeated malaria infection often stimulates polyclonal production of non-specific IgG [58]. These factors may also affect the amount and timing of transplacental transfer ICs to the fetus.

Thus, the wealth of studies to date, especially in humans, demonstrate that specific receptor-mediated transfer of IgG immunoproteins is regulated by the transporter/receptor expression not only on the syncytiotrophoblast but also on fetal endothelium and stromal tissues. Further, the Fc end of the immunoglobulin defines the ability to be transferred [59], and growing evidence suggests that antigens bound to these immunoglobulins, e.g., malaria proteins, may frequently be transferred through the placenta and extraembryonic membranes to the fetus, much like the “Trojan horse” of the past. This exposure could have dramatic effects on the fetal immune response with several possible outcomes. Some offspring may acquire protective immune responses that persist into infancy. Alternatively, the fetus may acquire immune tolerance, either by anergy or deletion of T cell clones or the development of regulatory T cells [60], [61]. These latter responses may increase the infant's susceptibility to malaria infection and disease. Whether the infant acquires protection or tolerance may depend on the timing, or the amount and type of antigen to which the fetus is exposed during gestation. An understanding of these outcomes would have important implications as to when, and how much, malaria chemoprophylaxis should be administered during pregnancy in endemic areas.
